# Predictors of false−negative serum thyroglobulin in persistent/recurrent Papillary Thyroid Carcinoma cervical lymph nodes

**DOI:** 10.3389/fonc.2026.1869134

**Published:** 2026-06-17

**Authors:** Jing Lin, Alibiyati Aini, Zien Qin

**Affiliations:** 1 Department of General Surgery, Cancer Hospital of Xinjiang Medical University, Urumqi, Xinjiang, China; 2 Division of Thyroid Surgery, General Surgery Department, Xiangya Hospital, Central South University, Changsha, Hunan, China; 3 Department of Thyroid and Breast Surgery, LiXian People’s Hospital, Changde, Hunan, China

**Keywords:** lymph nodes, persistent and recurrent disease, thyroglobulin, thyroglobulin antibody, thyroid cancer

## Abstract

**Purpose:**

Serum thyroglobulin (Tg) is the primary biomarker for monitoring disease persistence and recurrence in papillary thyroid carcinoma (PTC). However, some patients with structurally confirmed persistent/recurrent cervical lymph nodes (LNs) have low or undetectable Tg. This study aimed to identify predictors of false−negative unstimulated serum Tg (<0.2 ng/mL) and develop a predictive model.

**Patients and methods:**

This retrospective study enrolled 215 Papillary Thyroid Carcinoma patients who underwent total thyroidectomy and were confirmed to have persistent/recurrent cervical LNs. Multivariable logistic regression identified independent predictors associated with serum Tg <0.2 ng/mL. A nomogram was developed and validated by AUC, calibration curve, decision curve analysis (DCA).

**Results:**

Among the 215 patients (50 persistent, 165 recurrent), 48 (22.3%) had unstimulated serum Tg <0.2 ng/mL. Independent predictors of false−negative Tg (<0.2 ng/mL) were central LN compartment *(p* = 0.039), smaller LN size (*p* = 0.019), and higher Tg−Ab level (*p* < 0.001). In Tg−Ab negative patients (≤115 IU/mL, n=158), higher Tg−Ab remained associated with false−negative Tg (*p* < 0.001). The nomogram showed good discrimination (AUC = 0.87) and calibration. DCA demonstrated net clinical benefit at a threshold ≥0.1, defining high risk for false−negative Tg.

**Conclusion:**

Central LN location, small LN size, and elevated Tg−Ab predict false−negative Tg in Papillary Thyroid Carcinoma patients with persistent/recurrent LNs. The nomogram identifies high−risk individuals (≥0.1), in whom serum Tg alone is unreliable and adjunct imaging and biopsy should be considered.

## 1 Introduction

With the widespread application of high-resolution imaging and improved detection methods, the incidence of thyroid cancer has increased rapidly worldwide, making it one of the fastest-growing malignancies (1–3). Papillary thyroid carcinoma (PTC) is the most common histological subtype, accounting for the majority of cases (4, 5). Although Papillary Thyroid Carcinoma is generally regarded as an indolent tumor with favorable prognosis, persistent or recurrent disease occurs in approximately 1.4% to 9% of patients after initial treatment, posing a practical challenge to long-term management and quality of life (6–9). Accurate evaluation and early intervention for persistent/recurrent disease are therefore critically important.

Serum thyroglobulin (Tg) is produced by thyroid follicular cells and Papillary Thyroid Carcinoma cells and has become the primary method to evaluate and monitor persistent and recurrent diseases in Papillary Thyroid Carcinoma patients (10, 11). Based on robust evidence, the 2015 American Thyroid Association (ATA) guidelines proposed that postoperative unstimulated serum Tg levels greater than 0.2 ng/mL after total thyroidectomy was an independent predictor of persistent or recurrent diseases, and these Papillary Thyroid Carcinoma patients will likely need additional evaluations and therapies (12).

Despite its widespread use and clinical utility, serum Tg has notable limitations. A substantial number of Papillary Thyroid Carcinoma patients with structurally confirmed persistent or recurrent disease, particularly those with cervical lymph node (LN) metastases, present with unstimulated Tg levels below the 0.2 ng/mL threshold, leading to false-negative results and delayed intervention (13–15). Several factors have been proposed to contribute to this phenomenon, including the location and volume of recurrent LNs, as well as interference from endogenous thyroglobulin antibodies (Tg-Ab) (16–18). However, previous studies have largely focused on single factors or have been limited by small sample sizes, and the independent and combined effects of these factors remain incompletely understood.

Therefore, this study aimed to systematically investigate the factors influencing the predictive value of serum Tg for cervical persistent and recurrent LNs in a relatively large cohort of Papillary Thyroid Carcinoma patients who underwent reoperation. Specifically, we evaluated the impact of LN location (central vs. lateral compartment), LN volume, and Tg-Ab status on the diagnostic performance of serum Tg, with the goal of identifying patient subgroups in whom serum Tg is less reliable and alternative surveillance strategies may be needed.

## 2 Materials and methods

### 2.1 Patients and study design

This retrospective study reviewed Papillary Thyroid Carcinoma patients who underwent total thyroidectomy at Xiangya Hospital (January 2010–December 2024). We identified 470 patients who required reoperation for histopathology−confirmed persistent or recurrent cervical lymph node (LN) metastases. Persistent disease was defined as residual lesions confirmed within 6 months after initial surgery; recurrent disease as new lesions after ≥6 months of complete remission.

All suspicious LNs detected on imaging (ultrasound or CT) were confirmed by ultrasound−guided fine−needle aspiration (FNA). Most patients underwent selective LN dissection; a minority with solitary, small−volume (≤10 mm) low−risk LNs received radiofrequency ablation (RFA), with diagnosis based on pre−ablation FNA cytology.

**Figure 1:**
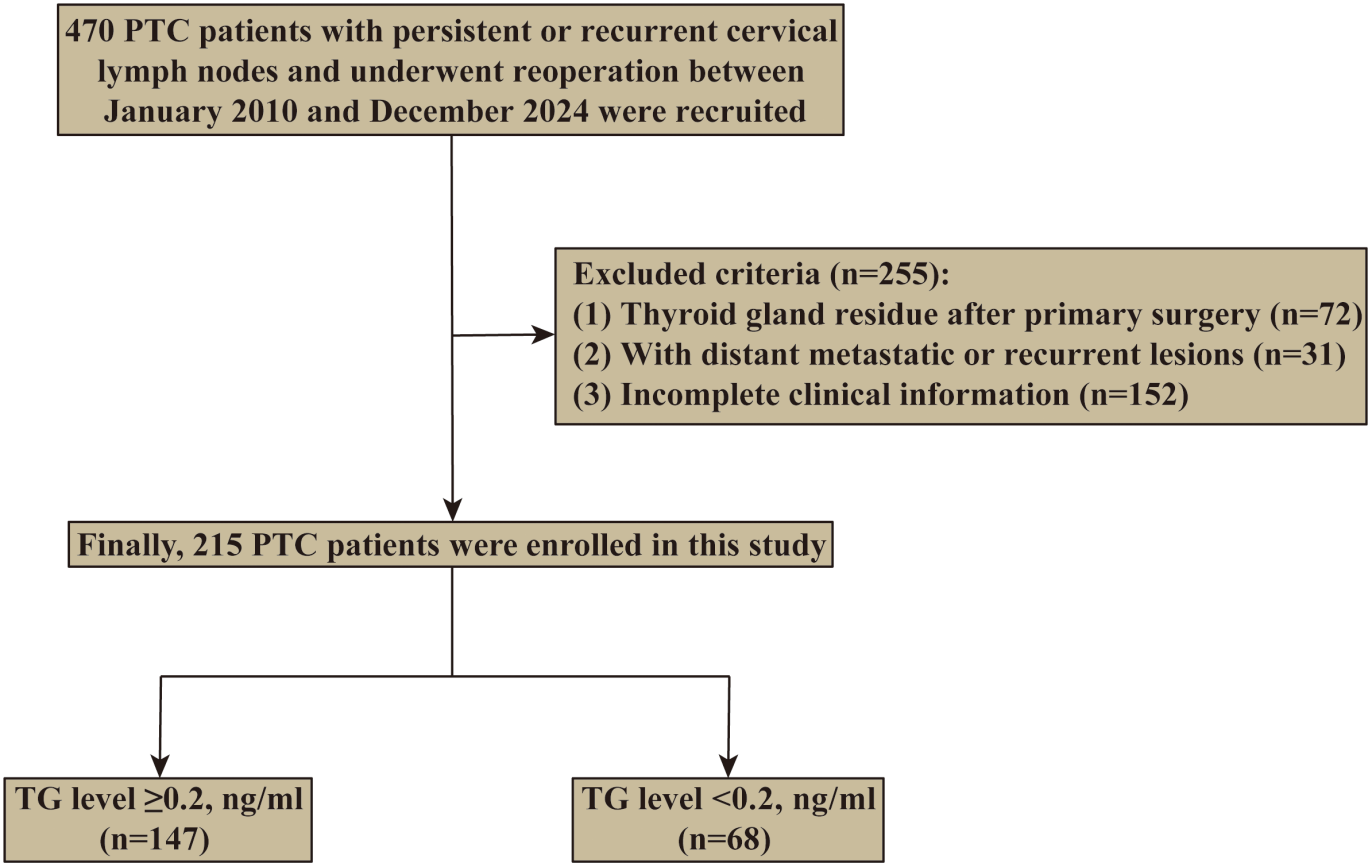
The flowchart of the inclusion and exclusion criteria for the study.

Exclusion criteria: (1) macroscopic residual thyroid tissue at reoperation (n=72); (2) distant metastasis (n=31); (3) incomplete data (n=152). Finally, 215 patients were included. The study flowchart summarizing patient selection is presented in **Figure 1**. The study was approved by the Ethics Review Committee of Xiangya Hospital (No. 2025121504). Informed consent was waived due to retrospective anonymized data.

### 2.2 Evaluation of clinicopathologic characteristics

All patients received thyrotropin (TSH) suppression therapy after total thyroidectomy, aiming to maintain TSH levels below 0.1 mIU/L for high-risk patients or within the lower normal range for others, according to the 2015 ATA guidelines. Postoperative surveillance included thyroid function tests and high−resolution neck ultrasound (US) every 6 months for the first 5 years, and annually thereafter, to evaluate for persistent or recurrent disease.

Serum free T3, free T4, TSH, Tg−Ab (reference 0–115 IU/mL), and TPO−Ab (0–34 IU/mL) were measured by electrochemiluminescence immunoassay (Roche). Unstimulated serum Tg ≥0.2 ng/mL was considered positive per ATA guidelines. For patients with elevated Tg (≥0.2 ng/mL) but negative ultrasound, contrast−enhanced CT was performed. LN size was defined as the maximal diameter of the largest metastatic LN on ultrasound (or CT when inconclusive). For patients with multiple metastatic LNs, the largest LN was used for size analysis.

### 2.3 Development and validation of the predictive model

Based on three independent predictors (LN location, LN size, Tg-Ab level), we developed a nomogram to estimate the individual risk of false-negative Tg (<0.2 ng/mL). Model discrimination was assessed by AUC; calibration was evaluated with a calibration curve using 1000 bootstrap resamples. Decision curve analysis (DCA) and clinical impact curve were used to assess clinical net benefit and application value. The risk threshold of 0.1 was selected based on the point where the model’s net benefit began to exceed that of the “treat-all” strategy on DCA.

### 2.4 Statistical analysis

Categorical variables were presented as frequencies and percentages (%), and differences between groups were compared using the chi-square or Fisher’s exact test, as appropriate. Continuous variables were expressed as median with interquartile range (IQR) and analyzed by Mann–Whitney U test.

Sensitivity and false negative rate were calculated using histopathologically confirmed LNs metastasis as the reference standard. LN size was also categorized into three clinically relevant groups (≤10 mm, >10–20 mm, >20 mm) based on the 2015 ATA guidelines (12). Tg-Ab was categorized as ≤57.5, >57.5–115, or >115 IU/mL. The 115 IU/mL cutoff is the manufacturer’s upper normal limit; the 57.5 IU/mL cutoff is the median Tg-Ab among detectable normal-range levels. In multivariable logistic regression, LN size (per 1 mm) and Tg-Ab (per 1 IU/mL) were treated as continuous variables to preserve statistical efficiency. A subgroup analysis was also performed in Tg-Ab-negative patients (≤115 IU/mL) to identify factors associated with false-negative Tg (<0.2 ng/mL).

Univariable and multivariable logistic regression identified factors influencing the predictive value of serum Tg for persistent/recurrent LNs. Variables with *p* <0.10 in univariable analysis were entered into the multivariable model. Results are reported as odds ratios (ORs) with 95% confidence intervals (CIs). All analyses were performed using SPSS 26.0 and GraphPad Prism 9.5.0. A two-sided *p* <0.05 was considered statistically significant.

## 3 Results

### 3.1 Comparison of clinicopathological characteristics of the candidates

A total of 215 Papillary Thyroid Carcinoma patients were included, consisting of 167 (77.7%) with serum Tg ≥0.2 ng/mL (Tg-positive) and 48 (22.3%) with serum Tg <0.2 ng/mL (Tg-negative). The cohort had a median age of 40 years (IQR 33–51), with 184 patients (85.6%) younger than 55 years; 68 (31.6%) were male and 147 (68.4%) females. Persistent lymph nodes (LNs) were present in 50 patients (23.3%) and recurrent LNs in 165 (76.7%). Lesions were detected by ultrasound alone in 186 patients (86.5%), by CT alone in 3 (1.4%), and by both in 26 (12.1%). Radioactive iodine ablation was administered to 87 patients (40.5%), with no significant difference between the Tg-positive and Tg-negative groups (38.9% vs. 45.8%, *p* = 0.390) (**Table 1**).

**Table 1:** Comparison of clinicopathologic characteristics among Tg positive and negative Papillary Thyroid Carcinoma patients with cervical persistent or recurrent lymph nodes.

Characteristics	Participants, no. (%)			P-value
	Total (n, %)	Tg positive (≥0.2, ng/ml)	Tg negative (<0.2, ng/ml)	
No. of patients	215 (100.0%)	167 (100.0%)	48 (100.0%)	
Sex				
Male	68 (31.6%)	55 (32.9%)	13 (27.1%)	0.442a
Female	147 (68.4%)	112 (67.1%)	35 (72.9%)	
Age at diagnosis				
Median (IQR, year)	40 (33 - 51)	40 (33 - 51)	39 (32 - 50)	0.644b
<55	184 (85.6%)	141 (84.4%)	43 (89.6%)	0.370a
≥55	31 (14.4%)	26 (15.6%)	5 (10.4%)	
RAI ablation				
Yes	87 (40.5%)	65 (38.9%)	22 (45.8%)	0.390a
No	128 (59.5%)	102 (61.1%)	26 (54.2%)	
Detected imaging methods for LNs				
US	186 (86.5%)	140 (83.8%)	46 (95.8%)	0.096a
CT	3 (1.4%)	3 (1.8%)	0 (0.0%)	
Both	26 (12.1%)	24 (14.4%)	2 (4.2%)	
Residual / recurrent LNs				
Residue	50 (23.3%)	38 (22.8%)	12 (25.0%)	0.746a
Recurrence	165 (76.7%)	129 (77.2%)	36 (75.0%)	
Residual / recurrent LNs location				
Central compartment	22 (10.2%)	11 (6.6%)	11 (22.9%)	0.003a
Lateral compartment	156 (72.6%)	124 (74.3%)	32 (66.7%)	
Central and lateral compartment	37 (17.2%)	32 (19.2%)	5 (10.4%)	
Residual / recurrent LNs number				
Median (IQR)	3 (1 - 6)	3 (2 - 6)	3 (1 - 6)	0.138b
Residual / recurrent LNs size				
Median (IQR, mm)	15 (11 - 20)	16 (12 - 21)	12 (10 - 16)	0.003b
Follow-up time				
Median (IQR, months)	13 (6 - 31)	13 (6 - 32)	17 (5 - 31)	0.950b
fT3 level, Median (IQR, pmol/l)	4.75 (4.24 - 5.39)	4.74 (4.22 - 5.43)	4.82 (4.27 - 5.39)	0.719b
fT4 level, Median (IQR, pmol/l)	20.27 (17.96 - 22.85)	20.12 (17.64 - 23.11)	20.69 (18.32 - 22.08)	0.776b
TSH level, Median (IQR, mIU/l)	0.32 (0.04 - 1.62)	0.37 (0.05 - 1.92)	0.16 (0.02 - 1.04)	0.121b
TPO-Ab level, Median (IQR, IU/ml)	15.16 (9.36 - 29.97)	14.10 (8.89 - 23.98)	32.77 (12.51 - 63.85)	<0.001b
Tg-Ab level, Median (IQR, IU/ml)	19.51 (13.33 - 124.10)	15.89 (12.65 -24.70)	451.75 (72.31 - 1105.20)	<0.001b

LNs, lymph nodes; Papillary Thyroid Carcinoma, papillary thyroid cancer; RAI, radioactive Iodine; fT3, free triiodothyronine; fT4, free thyroxine; TSH, thyrotropin; Tg, Thyroglobulin; Tg-Ab, thyroglobulin antibody; TPO-Ab, thyroid peroxidase antibody.

Variables with statistical significance are shown in bold; ^a^ Chi-square test; ^b^ Mann-Whitney U test.

Compared with Tg-positive patients, those with false−negative Tg (<0.2 ng/mL) had a significantly higher proportion of LNs located in the central compartment (22.9% vs. 6.6%, *p* = 0.003), smaller LN size (median 12 mm vs. 16 mm, *p* = 0.003), and higher median levels of TPO-Ab (32.77 vs. 14.10 IU/mL, *p* < 0.001) and Tg-Ab (451.75 vs. 15.89 IU/mL, *p* < 0.001) (**Table 1**).

### 3.2 Factors influencing serum Tg level in Papillary Thyroid Carcinoma patients with cervical persistent and recurrent LNs

**Figure 2:**
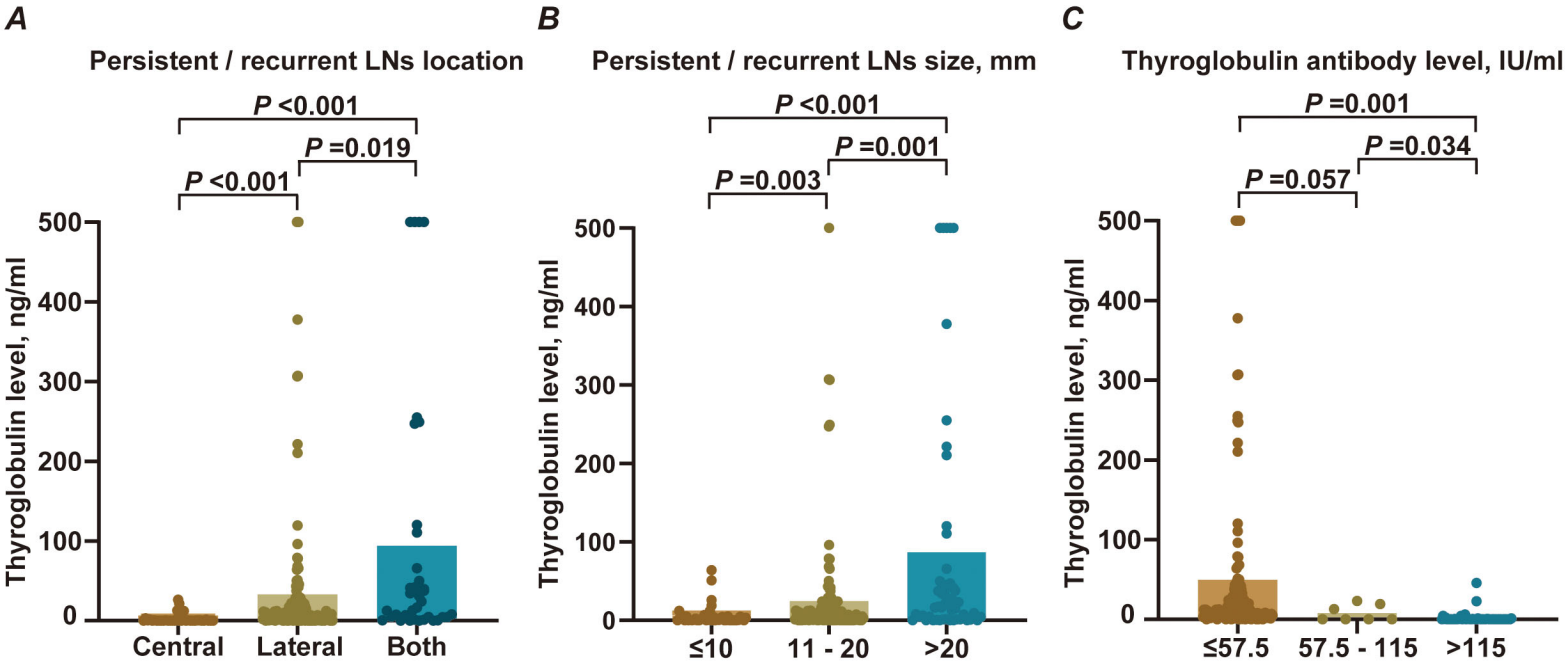
The difference of serum Tg level of the Papillary Thyroid Carcinoma patients with persistent and recurrent lymph nodes. **(A)** Persistent and recurrent LNs location. **(B)** Persistent and recurrent LNs volume. **(C)** Thyroglobulin antibody level. **Figure 2**. | Association of clinicopathological factors with serum Tg level in Papillary Thyroid Carcinoma patients having persistent or recurrent lymph nodes. **(A)** Lymph node location; **(B)** Lymph node size; **(C)** Thyroglobulin antibody (TgAb) level.

Multivariable logistic regression analysis was performed to identify independent factors associated with false-negative serum Tg (<0.2 ng/mL). As shown in **Table 2**, LN location (*p* = 0.039), LN size (*p* = 0.019), and Tg-Ab level (*p* < 0.001) were independent predictors of false-negative Tg. Specifically, compared with central compartment LNs, lateral compartment LNs (OR = 0.312, 95% CI 0.112–0.873, *p* = 0.026) and combined central/lateral LNs (OR = 0.180, 95% CI 0.042–0.778, *p* = 0.022) were associated with significantly lower odds of false-negative Tg. Smaller LN size (per 1 mm increase, OR = 0.930, 95% CI 0.875–0.988, *p* = 0.019) and higher Tg-Ab level (per 1 IU/mL increase, OR = 1.002, 95% CI 1.001–1.002, *p* < 0.001) were also independently associated with false-negative Tg. TPO-Ab level showed no independent association (*p* = 0.932). Of note, patients with central compartment LNs, smaller LN size, or higher Tg-Ab levels tended to have lower serum Tg levels (**Figure 2**).

**Table 2:** Multivariate logistic analysis of the independent factors associated with serum Tg level lower than 0.2 ng/ml in Papillary Thyroid Carcinoma patients with persistent or recurrent cervical lymph nodes.

Risk factor	Unadjusted		Multivariable adjusted	
	Odds ratio (95% CI)	P-value	Odds ratio (95% CI)	P-value
Residual / recurrent LNs location				
Central compartment	1 [Reference]	0.006	1 [Reference]	0.039
Lateral compartment	0.258 (0.103 - 0.649)	0.004	0.312 (0.112 - 0.873)	0.026
Central and lateral compartment	0.156 (0.044 - 0.551)	0.004	0.180 (0.042 - 0.778)	0.022
Residual / recurrent LNs size, Median (IQR, mm)	0.947 (0.901 - 0.996)	0.033	0.930 (0.875 - 0.988)	0.019
TPO-Ab level, Median (IQR, IU/ml)	1.003 (1.000 - 1.007)	0.050	1.000 (0.996 - 1.004)	0.932
Tg-Ab level, Median (IQR, IU/ml)	1.001 (1.001 - 1.002)	<0.001	1.002 (1.001 - 1.002)	<0.001

LNs, lymph nodes; Papillary Thyroid Carcinoma, papillary thyroid cancer; Tg, Thyroglobulin; Tg-Ab, thyroglobulin antibody; TPO-Ab, thyroid peroxidase antibody.

Variables with statistical significance are shown in bold.

### 3.3 Subgroup analysis in patients with Tg−Ab within the normal range (≤115 IU/mL)

We further analyzed the subgroup of patients with negative Tg−Ab (≤115 IU/mL, n=158) to assess whether tumor−related factors independently predict false−negative serum Tg after excluding overt antibody interference. Among these 158 patients, 13 (8.2%) had false−negative unstimulated serum Tg (<0.2 ng/mL) despite histopathologically confirmed persistent/recurrent cervical LNs, while 145 had true−positive Tg (≥0.2 ng/mL). The clinicopathological characteristics and regression results are summarized in **Supplementary Table 1**.

In univariable analysis, compared with central compartment location, lateral compartment location (OR 0.120, 95% CI 0.032–0.452, *p* = 0.002) and smaller LN size (per 1 mm increase, OR 0.882, 95% CI 0.787–0.987, *p* = 0.029) were associated with lower odds of false−negative Tg, whereas higher Tg−Ab level (per 1 IU/mL increase, OR 1.064, 95% CI 1.033–1.097, *p* < 0.001) was associated with higher odds (**Supplementary Table 2**). Multivariable analysis was not performed because the limited number of false-negative events (n=13) would lead to an overfitted model and unreliable estimates.

### 3.4 Diagnostic efficacy of serum Tg for cervical persistent/recurrent LNs by LN location, size, and Tg−Ab level

Next, we evaluated the impact of related factors on the diagnostic efficacy of serum Tg for cervical persistent/recurrent LNs. As shown in **Table 3**, the sensitivity of serum Tg decreased and FNR increased progressively with central compartment location (sensitivity 50.0%, FNR 50.0%), smaller LN size (≤10 mm: 65.1% and 34.9%; >10–20 mm: 78.3% and 21.7%; >20 mm: 86.5% and 13.5%), and higher Tg−Ab levels (≤57.5 IU/mL: 94.0% and 6.0%; >57.5–115 IU/mL: 42.9% and 57.1%; >115 IU/mL: 38.6% and 61.4%).

**Table 3:** The diagnostic efficacy of serum Tg level ≥0.2 ng/ml for cervical persistent or recurrent lymph nodes in Papillary Thyroid Carcinoma patients.

Risk factor	Sensitivity	FNR
Residual / recurrent LNs location		
Central compartment	50.0%	50.0%
Lateral compartment	79.5%	20.5%
Central and lateral compartment	86.5%	13.5%
Residual / recurrent LNs size, mm		
≤10	65.1%	34.9%
>10 and ≤20	78.3%	21.7%
>20	86.5%	13.5%
Thyroglobulin antibody level, IU/ml		
≤57.5	94.0%	6.0%
>57.5 and ≤115	42.9%	57.1%
>115	38.6%	61.4%

FNR, false-negative rate; Papillary Thyroid Carcinoma, papillary thyroid cancer; Tg, Thyroglobulin.

### 3.5 Diagnostic efficacy of the prediction model for false−negative Tg in Papillary Thyroid Carcinoma patients

**Figure 3:**
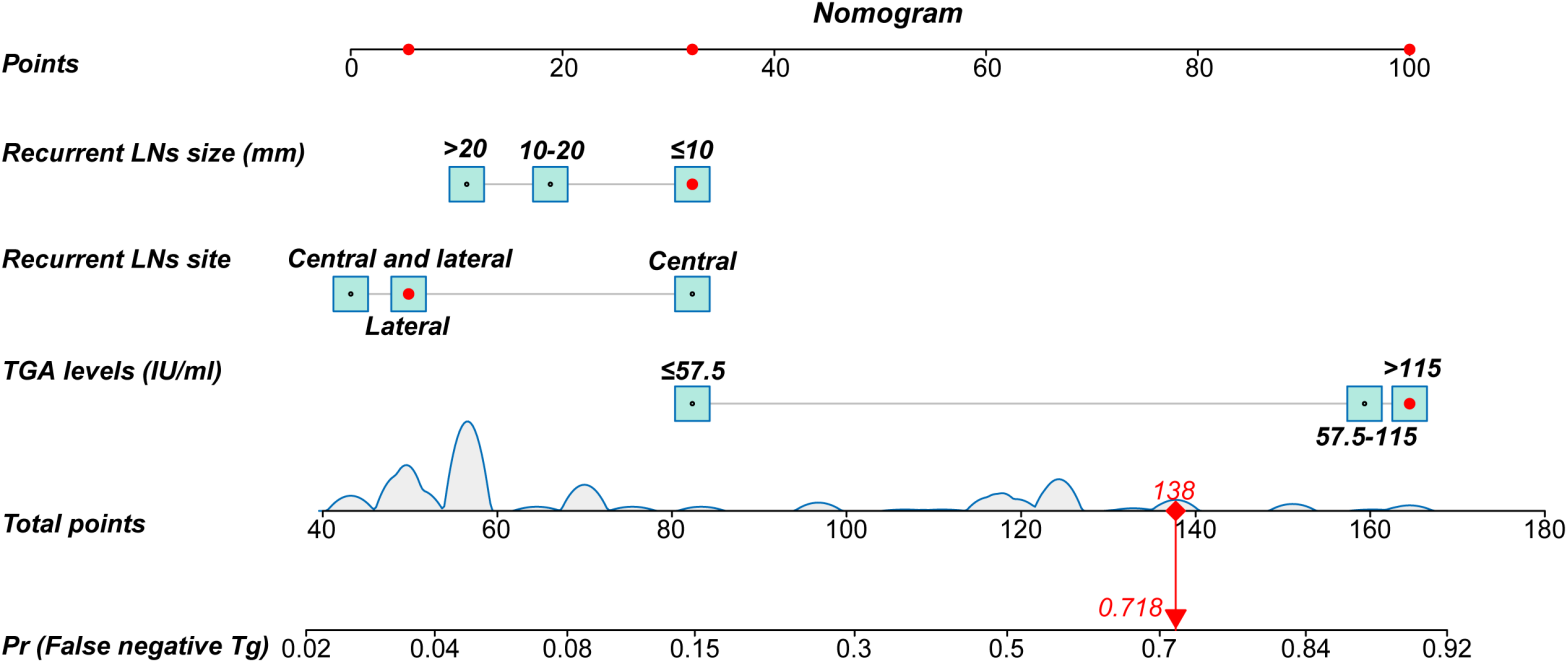
A nomogram for predicting false−negative Tg in Papillary Thyroid Carcinoma patients. **(A)** Lymph node location; **(B)** Lymph node size; **(C)** Thyroglobulin antibody (TgAb) level.

We developed a predictive model based on three independent factors (lymph node location, lymph node size, and Tg−Ab level) to estimate the risk of a false-negative serum Tg result (<0.2 ng/mL) in Papillary Thyroid Carcinoma patients with pathologically confirmed cervical persistent or recurrent lymph nodes (**Figure 3**). The model integrates these factors and calculates a total score; a higher total score indicates a greater likelihood of false-negative serum Tg.

**Figure 4:**
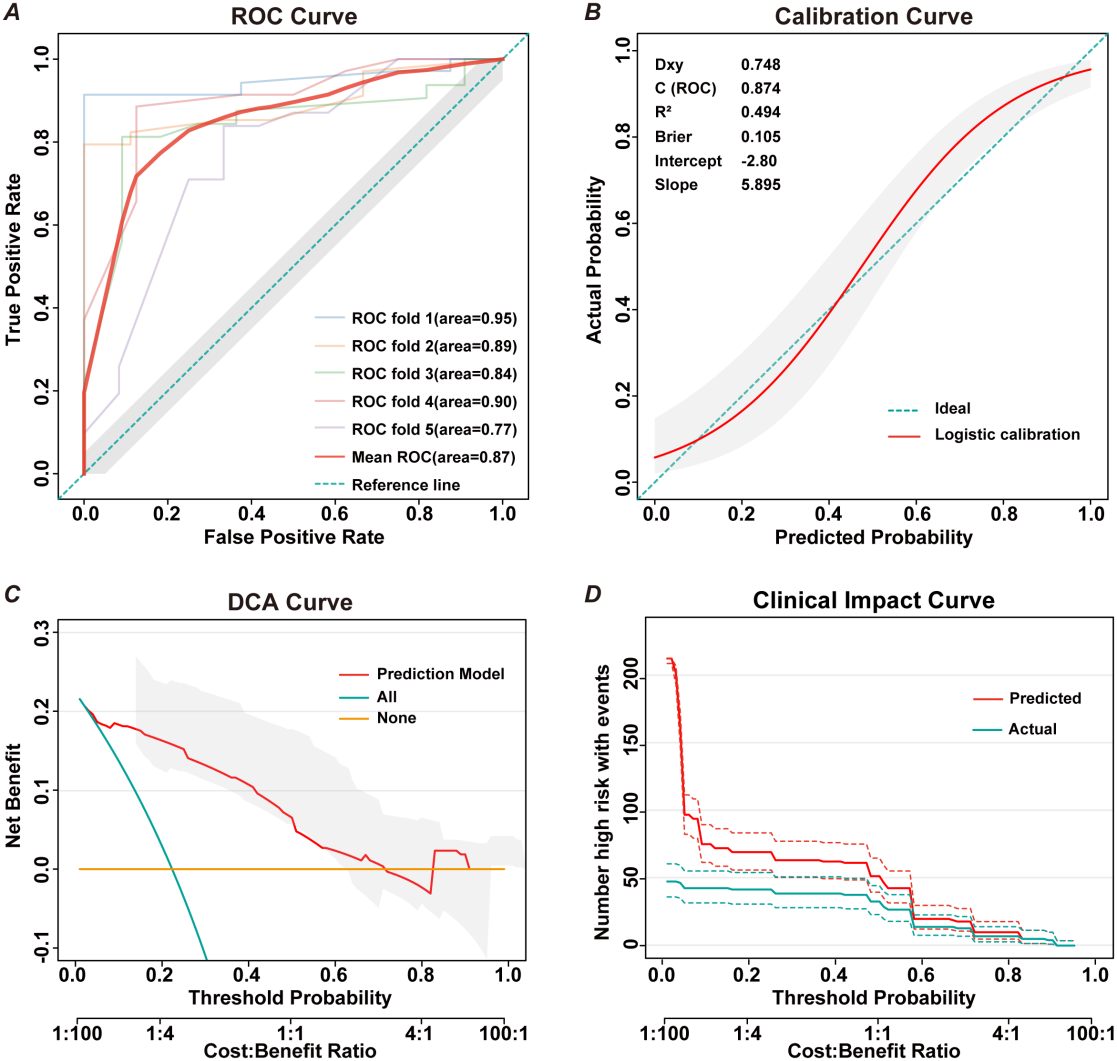
Validation of the nomogram. **(A)** ROC curve; **(B)** Calibration curve; **(C)** Decision curve analysis (DCA) curve; **(D)** Clinical impact curve.

The model was validated using multiple methods (**Figure 4**). The ROC curve showed excellent discriminative ability, with an AUC of 0.87 (**Figure 4A**). The calibration curve demonstrated good agreement between the predicted probability and the observed outcomes (**Figure 4B**). Decision curve analysis (DCA) revealed that the model’s net benefit began to exceed that of the “treat-all” strategy at a threshold probability of approximately 0.1, and the net benefit increased substantially thereafter (**Figure 4C**). Accordingly, patients with a predicted probability ≥0.1 were classified as high risk for false−negative Tg, and those with <0.1 as low risk. The clinical impact curve further supported this threshold (**Figure 4D**).

## 4 Discussion

Persistent or recurrent lesions significantly affect postoperative quality of life and prognosis in Papillary Thyroid Carcinoma patients. Serum Tg, produced by thyroid follicular cells and Papillary Thyroid Carcinoma cells, has become the primary biomarker for monitoring persistent and recurrent disease (10, 19). However, postoperative serum Tg is often undetectable in Papillary Thyroid Carcinoma patients with established persistent/recurrent disease, which may delay early evaluation and clinical management (13–15). Therefore, we comprehensively analyzed factors influencing the diagnostic value of serum Tg for persistent/recurrent cervical LNs in Papillary Thyroid Carcinoma.

In this study, central compartment location, smaller lymph node (LN) size, and higher thyroglobulin antibody (Tg-Ab) levels were independently associated with false-negative serum Tg (<0.2 ng/mL) in Papillary Thyroid Carcinoma patients with surgically confirmed persistent/recurrent cervical LN metastases. These factors substantially compromised the diagnostic sensitivity of serum Tg, raising the risk of delayed intervention. Based on these predictors, we developed a predictive model to estimate individual risk of false−negative Tg. Using a risk threshold of 0.1 derived from decision curve analysis, patients with a predicted probability ≥0.1 are classified as high risk—in whom serum Tg alone is unreliable and ultrasound combined with fine−needle aspiration is recommended—whereas those with probability <0.1 are low risk, allowing serum Tg to be used reliably for clinical decision−making.

Several mechanisms may explain these findings. First, central compartment recurrences often present with smaller or more indolent tumor burdens, resulting in lower serum Tg levels. This is consistent with our observation that central compartment LNs are generally smaller and aligns with previous reports (20). Second, the well−established relationship between tumor burden and Tg secretion indicates that smaller volume metastases produce less Tg, which may fall below the detection threshold of standard immunometric assays. Bachelot et al. demonstrated that total metastatic LN volume significantly correlates with serum Tg levels, and undetectable Tg should not be considered a reliable criterion to exclude minimal residual disease (21). Third, Tg−Ab interference is a recognized cause of falsely low or undetectable Tg values, as endogenous antibodies can bind Tg and block its detection in immunoassays (22).

Our results are consistent with previous studies showing that positive Tg−Ab impairs the diagnostic performance of serum Tg for cervical persistent/recurrent LNs in Papillary Thyroid Carcinoma patients (23, 24). However, to our knowledge, this is one of the few studies to systematically evaluate the combined and independent effects of LN location, LN size, and Tg−Ab status within a single cohort, and to integrate these factors into a clinically applicable predictive model. For instance, in our cohort, positive Tg−Ab was associated with a low sensitivity (38.6%) and a high false−negative rate (FNR, 61.4%) for detecting persistent/recurrent LNs. Similarly, serum Tg showed poor performance for central compartment LNs (sensitivity 50.0%, FNR 50.0%) and for LNs ≤10 mm (sensitivity 65.1%, FNR 34.9%). These findings suggest that reliance on serum Tg alone may lead to underestimation of disease burden and potentially delayed surgical intervention.

Of particular interest, our subgroup analysis restricted to patients with Tg−Ab levels within the normal range (≤115 IU/mL) revealed that even among these ‘negative’ patients, higher Tg−Ab values independently predicted false−negative serum Tg (OR 1.064 per IU/mL, *p* < 0.001). This finding extends current knowledge by demonstrating that Tg−Ab interference is not an all−or−none phenomenon; even sub−threshold Tg−Ab concentrations can affect the accuracy of Tg immunoassays. Clinically, a Tg−Ab result within the reference range should not be overinterpreted as a guarantee of no interference, especially when serum Tg is unexpectedly low in the presence of suspicious imaging findings.

Our findings suggest that in Papillary Thyroid Carcinoma patients with postoperative undetectable or low serum Tg (<0.2 ng/mL) who have risk factors such as central compartment recurrence, small LNs (≤10 mm), or positive (or even borderline−high) Tg−Ab levels, clinicians should not rely solely on serum Tg to rule out persistent/recurrent disease. In these high−risk subgroups, closer surveillance with high−resolution neck ultrasound—and, if indicated, cross−sectional imaging or ultrasound−guided fine−needle aspiration biopsy—is warranted. A normal Tg level should be interpreted with caution, particularly during the first 2−3 years after total thyroidectomy when most recurrences occur. Our predictive model provides a quantitative tool to identify patients who would benefit from such intensified monitoring.

This study has several limitations inherent to its retrospective, single−center design. First, while the events−per−variable (EPV) of 16 (48 events/3 predictors) was adequate for main−effect estimation, the sample size was insufficient to reliably detect higher−order interactions (e.g., between LN size and Tg−Ab). Therefore, our nomogram should be interpreted as a main−effects model, and external validation in larger cohorts is needed. Second, the exclusion of 152 patients due to incomplete clinical data may have introduced selection bias. Third, the sample size—particularly the small number of false−negative events (n=13 in the Tg−negative group)—limited our ability to perform more complex multivariable modeling or detailed subgroup−by−subgroup analyses. Fourth, we did not evaluate dynamic changes in Tg over time, TSH suppression, or RAI as time−varying covariates. Finally, because some pathological subtypes of Papillary Thyroid Carcinoma (e.g., tall cell, columnar cell) are rare and not routinely reported in all centers, we could not assess whether subtype−specific differences in Tg expression exist.

## 5 Conclusion

In conclusion, in Papillary Thyroid Carcinoma patients with postoperative undetectable or low serum Tg (<0.2 ng/mL), the presence of central compartment recurrence, small LNs (≤10 mm), or positive/elevated Tg−Ab levels should alert clinicians to the unreliability of serum Tg alone for excluding persistent/recurrent disease. Enhanced surveillance with neck ultrasound and, where appropriate, fine−needle aspiration is recommended. Future prospective, multicenter studies with standardized Tg assay protocols and recovery testing are needed to validate our predictive model for false−negative Tg and to further clarify the independent contributions of tumor−related factors.

## Data Availability

The raw data supporting the conclusions of this article will be made available by the authors, without undue reservation.
