# Supplementary material for: Predictors of false−negative serum thyroglobulin in persistent/recurrent Papillary Thyroid Carcinoma cervical lymph nodes
Source: Front Oncol. 2026 Jun 17;16:1869134. doi: 10.3389/fonc.2026.1869134 (PMC13318710; doi:10.3389/fonc.2026.1869134)
Supplement: Supplementary file 1 [file Table1.docx]

Supplementary Material

| **Supplementary Table 1. Clinicopathologic characteristics of TgAb-negative PTC patients stratified by serum Tg level (≥0.2 vs. <0.2 ng/mL).** | | | | |
| --- | --- | --- | --- | --- |
| **Characteristics** | **Participants, No. (%)** | | | ***p-*value** |
|  | **Total**  **(n, %)** | **Tg Positive**  **(****≥0.2, ng/ml)** | **Tg Negative**  **(****<0.2, ng/ml)** |  |
| No. of patients | 158 (100.0%) | 145 (100.0%) | 13 (100.0%) |  |
| Sex |  |  |  |  |
| Male | 52 (32.9%) | 50 (34.5%) | 2 (15.4%) | 0.273^a^ |
| Female | 106 (67.1%) | 95 (65.5%) | 11 (84.6%) |  |
| Age at diagnosis |  |  |  |  |
| Median (IQR, year) | 42 (33 - 51) | 40 (33 - 51) | 43 (37 - 54) | 0.597^b^ |
| <55 | 132 (83.5%) | 121 (83.4%) | 11 (84.6%) | 0.999^a^ |
| ≥55 | 26 (16.5%) | 24 (16.6%) | 2 (15.4%) |  |
| RAI ablation |  |  |  |  |
| Yes | 57 (36.1%) | 53 (36.6%) | 4 (30.8%) | 0.771^a^ |
| No | 101 (63.9%) | 92 (63.4%) | 9 (69.2%) |  |
| Detected imaging methods for LNs |  |  |  |  |
| US | 134 (84.8%) | 121 (83.4%) | 13 (100.0%) | 0.281^a^ |
| CT | 3 (1.9%) | 3 (2.1%) | 0 |  |
| Both | 21 (13.3%) | 21 (14.5%) | 0 |  |
| Residual / recurrent LNs |  |  |  |  |
| Residue | 29 (18.4%) | 28 (19.3%) | 1 (7.7%) | 0.508^a^ |
| Recurrence | 129 (81.6%) | 117 (80.7%) | 12 (92.3%) |  |
| Residual / recurrent LNs location |  |  |  |  |
| Central compartment | 13 (8.2%) | 8 (5.5%) | 5 (38.5%) | <0.001^a^ |
| Lateral compartment | 115 (72.8%) | 107 (73.8%) | 8 (61.5%) |  |
| Central and lateral compartment | 30 (19.0%) | 30 (20.7%) | 0 |  |
| Residual / recurrent LNs number |  |  |  |  |
| Median (IQR) | 3 (1 - 6) | 3 (1 - 6) | 2 (1 - 4) | 0.081^b^ |
| Residual / recurrent LNs size |  |  |  |  |
| Median (IQR, mm) | 16 (11 - 21) | 16 (12 - 21) | 12 (9 - 16) | 0.020^b^ |
| Follow-up time |  |  |  |  |
| Median (IQR, months) | 14 (6 - 34) | 13 (6 - 35) | 23 (8 - 36) | 0.148^b^ |
| fT3 level, Median (IQR, pmol/l) | 4.73 (4.22 - 5.51) | 4.75 (4.22 - 5.53) | 4.42 (4.15 - 5.33) | 0.412^b^ |
| fT4 level, Median (IQR, pmol/l) | 20.65 (18.00 - 23.40) | 20.51 (17.99 - 23.46) | 20.81 (17.91 - 23.86) | 0.772^b^ |
| TSH level, Median (IQR, mIU/l) | 0.29 (0.04 - 1.74) | 0.34 (0.05 - 1.77) | 0.11 (0.02 - 1.80) | 0.385^b^ |
| TPO-Ab level, Median (IQR, IU/ml) | 12.66 (7.73 - 21.40) | 13.42 (8.00 – 21.02) | 11.77 (6.55 - 29.89) | 0.384^b^ |
| Tg-Ab level, Median (IQR, IU/ml) | 15.40 (12.57 - 22.37) | 15.07 (12.55 -19.85) | 51.18 (28.04 – 59.68) | <0.001^b^ |
| **Abbreviations:** LNs, lymph nodes; PTC, papillary thyroid cancer; RAI, radioactive Iodine; fT3, free triiodothyronine; fT4, free thyroxine; TSH, thyrotropin; Tg, Thyroglobulin; Tg-Ab, thyroglobulin antibody; TPO-Ab, thyroid peroxidase antibody. | | | | |
| **Note:** Variables with statistical significance are shown in bold; ^a^ Chi-square test; ^b^ Mann-Whitney U test. | | | | |

| **Supplementary Table 2.**  **Univariable logistic analysis of factors associated with serum Tg level lower than 0.2 ng/ml in TgAb-negative PTC patients with persistent or recurrent cervical lymph nodes.** | | |
| --- | --- | --- |
| **Characteristics** | **Unadjusted** | |
|  | **Odds Ratio**  **(95% CI)** | ***p-*value** |
| Residual / recurrent LNs location |  |  |
| Central compartment | 1 [Reference] |  |
| Lateral compartment | 0.120 (0.032 - 0.452) | 0.002 |
| Central and lateral compartment | NA | NA |
| Residual / recurrent LNs size, Median (IQR, mm) | 0.882 (0.787 - 0.987) | 0.029 |
| Tg-Ab level, Median (IQR, IU/ml) | 1.064 (1.033 - 1.097) | <0.001 |
| **Abbreviations:** LNs, lymph nodes; PTC, papillary thyroid cancer; Tg, Thyroglobulin; Tg-Ab, thyroglobulin antibody. | | |
| **Note:** Variables with statistical significance are shown in bold. | | |
